# Comparison Among Two Liquid Formulations of L-thyroxine in the Treatment of Congenital Hypothyroidism in the First Month of Life: A Pilot Study

**DOI:** 10.3389/fendo.2022.860775

**Published:** 2022-04-05

**Authors:** Gerdi Tuli, Jessica Munarin, Luisa de Sanctis

**Affiliations:** ^1^Department of Pediatric Endocrinology, Regina Margherita Children’s Hospital, Turin, Italy; ^2^Department of Health and Pediatric Sciences, Postgraduate Program in Biomedical Sciences and Oncology, University of Turin, Turin, Italy; ^3^Department of Health and Pediatric Sciences, Postgraduate School of Pediatrics, University of Turin, Turin, Italy; ^4^Department of Health and Pediatric Sciences, University of Turin, Turin, Italy

**Keywords:** congenital hypothyroidism, liquid L-thyroxine, newborns, pediatric age, treatment

## Abstract

The liquid formulation of L-thyroxine is the most used in the substitutive treatment of congenital hypothyroidism (CH). This formulation has higher TSH suppression rates with respect of L-thyroxine tablets and thus lower doses are indicated. Two types of liquid L-thyroxine (Tirosint^©^ and Tifactor^©^) are currently approved in Italy for use in pediatric age and to date there are no data available in the Literature comparing the two liquid formulations. The aim of this study is to compare the efficacy of both formulations in normalizing TSH and fT4 levels in the first month of life and to compare the L-thyroxine requirement for both formulations over the same period. All newborns diagnosed with primary CH at the neonatal screening program in the Piedmont region of Italy in the period May 2020 – May 2021 were enrolled and divided into two groups according to the liquid formulation used: TS Group with Tirosint^©^ and TF Group with Tifactor^©^. No difference was observed between the two groups considering the TSH at dried blood spot (DBS) at neonatal screening, the serum levels of TSH, fT4 and fT3 and initial dose of L-thyroxine. At 15 days the serum TSH in the TF Group was 0.08 ± 0.02 mcUI/ml, while in the TS Group it was 36.7 ± 14.7 mcUI/ml p=0.04). No differences were observed between the two groups considering fT4 levels and L-thyroxine requirement. Among the subjects in the TF Group, 5/9 showed suppressed TSH at 15 days after starting treatment, while none of the subjects in the TS Group showed TSH levels below the normal lower limit (p=0.011). Among the subjects in the TF Group, 5/9 patients showed suppressed TSH at 30 days after starting treatment, while 1/12 subjects in the TS Group showed TSH levels below the normal lower limit (p=0.017). In conclusion, this study confirms the efficacy in normalizing the thyroid hormonal profile in newborns with CH among the liquid solutions although the response seem to be different in timing therefore an individual approach is necessary considering the type of formulation used, the diagnostic category of CH and clinical features.

## Introduction

Congenital hypothyroidism (CH) is the most common endocrine disorder in pediatric age (1). Prompt diagnosis and treatment are fundamental to avoid neuro-developmental delay, thus neonatal screening strategies are implemented in most countries. In recent years, the incidence of CH has increased worldwide due to many factors such as the lowering of the TSH cut-off detection at neonatal screening, the increase of the neonatal population at risk for developing CH and genetic-environmental factors. This led to the change in incidence in the Piedmont region in Italy from 1:3000-4000 in the ‘90s and 1:2200 in the first decade of 2000 to the current 1:1090 (2). Once reported by the national screening center, the diagnosis of CH should be confirmed by evaluating the thyroid hormone profile of thyroid stimulating hormone (TSH), free Thyroxine (fT4) and free triiodothyronine (fT3), thyroglobulin dosage and radiological evaluation by ultrasound and/or Tc^99^ or I^121^ scintiscan. The titer of Anti-peroxidase (AbTPO), anti-thyroglobulin (AbHTG) and anti-TSH receptor antibodies should also always be determined at the time of diagnosis.

The latest guidelines indicate an initial dose of L-thyroxine at 10-15 mcg/kg/day, to be started within the first two weeks of life in case of severe CH and within the first month in milder conditions (1). Synthetic L-thyroxine has a comparable structure to T4, and the substitutive treatment can be started in tablet or liquid formulation (3). The solid formulation requires an acid gastric pH to be dissolved, thus its administration should take place 20-30 minutes before breakfast (4). The liquid preparation is bioequivalent to the solid formulation and does not need acid gastric pH for the dissolution phase due to its liquid physical state (5, 6).

Currently, the most used formulation in Italy in newborns is a liquid formulation administered in drops containing 3.57 mcg of L-thyroxine and 8.68 mg of ethanol (Tirosint^©^, Ibsa Farmaceutici, Lodi, Italia, approved in Italy since 2009). Since 2019 another L-thyroxine liquid formulation has been approved in Italy for use in the pediatric age (Tifactor^©^, Galenica Pharmaceutical Industry, Athens, Greece), containing 20 mcg of L-thyroxine in each ml and glycerol, but not ethanol. Chronic administration of ethanol does not represent a contraindication to prescribing Tirosint^©^ in the pediatric age but there is controversial concern about the potential risk of side effects from its assumption for a long period (7). Recent studies have observed a higher rate of TSH suppression in patients treated with the liquid formulation than in subjects treated with the solid formulation, exposing those patients to a greater risk of overtreatment, with iatrogenic hyperthyroidism, which may be harmful in the first years of life and increase the risk of developing attention disorders later in childhood (7–9). Therefore, some Authors indicate to use lower doses of the liquid formulation (Tirosint^©^) than the solid formulation (7).

To date, no data are available in the Literature comparing the two liquid formulations (Tirosint^©^ vs Tifactor^©^) approved for the treatment of hypothyroidism in pediatric age in Italy. The aim of this study is to compare the effectiveness of both formulations in normalizing the TSH and fT4 levels in the first month of life and to compare the L-thyroxine requirement for both formulations over the same period.

## Materials And Methods

All newborns diagnosed with primary CH, detected at the neonatal screening program in the Piedmont region in Italy in the period May 2020 – May 2021, were enrolled. All the TSH detection tests on dried blood spot (DBS) were performed at the regional reference center for Neonatal Screening at Regina Margherita Children’s Hospital, in Torino, Italy.

Infants with syndromic CH or chromosomal abnormalities, transient congenital hypothyroidism (TCH), or isolated hyperthyrotropinemia in which replacement treatment was not initiated have been excluded. Confirmation of the diagnosis of CH was based on serum TSH, fT4, fT3 and thyroglobulin levels, and on radiological evaluation with Tc99 scintigraphy and ultrasound examination in all cases of suspected thyroid agenesis. AbTPO and AbHTG antibodies were evaluated in case of unknown or positive maternal antibodies titer. All blood tests were performed in a single laboratory.

The patients were divided into two groups according to the liquid formulation used: TS Group using Tirosint^©^ and TF Group using Tifactor^©^. The choice of the liquid formulation was based on the discretion of the clinician and the wishes of the family. After the start of the treatment, both groups were evaluated according to the recent Guidelines, at 15 days and 1 month, by TSH and fT4 assays, as well as with the analysis of auxological parameters (weight, length, and head circumference).

Statistical analyses and graphs were performed through Graphpad 7 software (GraphPad Software, La Jolla, CA, USA), using T-student test to compare the means and the chi-square test to compare the differences between groups.

The study was performed according to the guidelines of the Declaration of Helsinki and received the approval of the Ethics Committee of the Hospital.

## Results

During the study period, 21 newborns (M=8, F=13) diagnosed with CH were enrolled. Demographic and clinical data are represented in **Table 1**.

**Table 1:** Demographic and clinical data of the 21 newborns with CH enrolled in the study.

	TF Group (n = 9)	TS Group (n = 12)	P value
Sex	M = 4 F= 5	M = 4 F= 8	0.60
Gestational age	39.4 ± 0.35	37.8 ± 0.68	0.08
Neonatal weight	3304 ± 168	2847 ± 181	0.08
Neonatal length	49.8 ± 0.7	48.1 ± 1.2	0.28
Congenital malformations	1/9	1/12	0.83
Comorbidities	4/9	6/12	0.80
Maternal thyroid disease	3/9	2/12	0.37
Type of CH:			
Eutopic	7	8	0.55
Ectopic	1	4	
Hypoplasia	1	0	

TF, Tifactor group; TS, Tirosint group.

Substitutive treatment with Tifactor was started in 9/21 patients (TF Group), while Tirosint was administered in 12/21 newborns (TS Group).

No differences were observed between the two groups for sex, gestational age, neonatal weight and length, congenital malformations, co-morbidities, and maternal thyroid disease.

In the TF Group, 7/9 newborns had diagnosis of CH with eutopic gland, while ectopic gland and thyroid hypoplasia were detected in the other 2 subjects, respectively.

In the TS Group, 8/12 newborns were affected by CH with eutopic gland, while in the other 4 subjects an ectopic gland was detected.

The thyroid hormone profile and L-thyroxine requirement are represented in **Table 2**. No difference was observed between the two groups considering the TSH levels at DBS (52.11 ± 23.8 mcUI/ml in the TF Group and 102.8 ± 26.8 mcUI7ml in the TS Group, p=.19) and TSH serum levels (95.68 ± 41.1 and 138.3 ± 37.3 mcUI/ml respectively, p=0.45). The same trend was also observed for serum fT4 (8.94 ± 1.28 and 8.46 ± 1.48 pg/ml respectively, p=0.81) and serum fT3 (3.97 ± 0.31 and 3.86 ± 0.48 pg/ml respectively, p=0.86). The initial L-thyroxine dose was 8.88 ± 1.05 mcg/kg/day in the TF Group and 9.78 ± 0.46 mcg/kg/day in the TS Group (p=0.4).

**Table 2:** Biochemical data and L-thyroxine requirement at diagnosis and after 15 and 30 days in the TF e TS groups.

	TF Group (n = 9)	TS Group (n = 12)	P value
**At diagnosis:**			
TSH DBS	52.11 ± 23.8	102.8 ± 26.8	0.19
TSH serum	95.68 ± 41.1	138.3 ± 37.3	0.45
fT4	8.94 ± 1.28	8.46 ± 1.48	0.81
fT3	3.97 ± 0.31	3.86 ± 0.48	0.86
L- thyroxine dose	8.88 ± 1.05	9.78 ± 0.46	0.40
**At 15 days:**			
TSH	0.08 ± 0.02	36.7 ± 14.7	**0.04**
FT4	21.6 ± 1.76	19.6 ± 1.68	0.42
L-thyroxine dose	7.75 ± 0.92	8.63 ± 0.61	0.42
Suppressed TSH	5/9	0/12	**0.011**
High fT4	5/9	6/12	0.80
**At 30 days:**			
TSH	0.48 ± 0.33	4.98 ± 3.34	0.26
FT4	20.1 ± 1.24	18.6 ± 1.29	0.44
L-thyroxine dose	5.98 ± 0.73	6.69 ± 0.63	0.47
Suppressed TSH	5/9	1/12	**0.017**
High fT4	5/9	3/12	0.15

TF, Tifactor group; TS, Tirosint group; DBS, Dry blood spot.

Significant results are in bold.

At 15 days, the serum TSH in the TF Group was 0.08 ± 0.02 mcUI/ml, while in the TS Group it was 36.7 ± 14.7 mcUI/ml p=0.04). No differences were observed between the two groups, considering fT4 levels (21.6 ± 1.76 and 19.6 ± 1.68 pg/ml respectively, p=0.42) and L-thyroxine requirement (7.75 ± 0.92 and 8.63 ± 0.61 mcg/kg/day respectively, p=0.42).

Among the subjects in the TF Group, 5/9 showed suppressed TSH at 15 days from the start of treatment, while none of the subjects in the TS Group showed TSH levels below the lower normal limit (p=0.011). High than normal fT4 serum levels were observed in 5/9 subjects in the TF group and 6/12 patients in the TS Group (p=0.8).

At 30 days, no differences were observed between the two groups considering serum TSH (0.48 ± 0.33 and 4.98 ± 3.34 mcUI/ml respectively, p=0.26), fT4 levels (20.1 ± 1.24 and 18.6 ± 1.29 pg/ml respectively, p=0.44) and L-thyroxine requirement (5.98 ± 0.73 and 6.69 ± 0.63 mcg/kg/day respectively, p=0.47).

Among the subjects in the TF Group, 5/9 patients showed suppressed TSH at 30 days after starting treatment, while 1/12 subjects in the TS Group showed TSH levels below the lower limit of normal (p=0.017). High than normal fT4 serum levels were observed in 5/9 subjects in the TF group and 3/12 patients in the TS Group (p=0.13).

**Figure 1:**
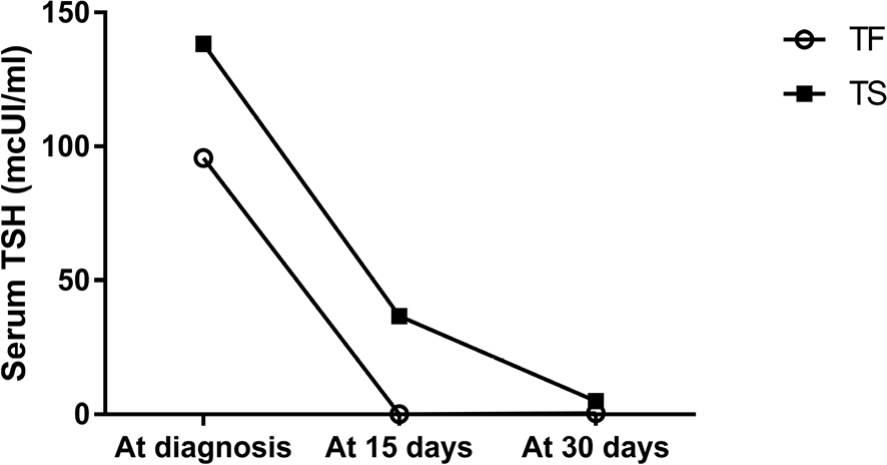
Serum TSH change using Tirosint and Tifactor in the first month of life in newborns affected by CH.

The mean TSH and fT4 levels and the mean L-thyroxine requirement at diagnosis and 15 and 30 days after the starting treatment have been represented in **Figure 1**. Subjects in the TF Group showed lower TSH levels, although statistically significant only at 15 days after starting treatment, higher fT4 levels and lower L-thyroxine dose requirement, although these parameters did not show any significant difference between the two groups.

## Discussion

Congenital hypothyroidism is the most common congenital endocrine disorder and the institution of prompt substitutive treatment in these newborns is critical to avoid the negative effects of hypothyroidism, particularly growth and neurodevelopment impairment. The liquid formulation of L-thyroxine is currently the most used in this population, given the greater bioavailability of the oral solution compared to the solid one. Furthermore, the liquid formulation does not require acid gastric pH for the dissolution phase compared to tablets.

Previous studies have established the effectiveness of the liquid formulation, also showing a greater risk of overtreatment during the first month, possibly due to a greater absorption of this formulation than tablets (7–9). Overtreatment should be avoided, especially in the first two years of life, as it may be associated to attention deficit hyperactivity disorder (ADHD), behavioral disorders and, if prolonged, cognitive impairment in the childhood (10, 11). Therefore, some authors suggest a lower dose at the start of treatment when using liquid formulations (7).

Currently in Italy two type of liquid L-thyroxine are approved for the paediatric age. Tirosint was approved since 2009 and it is administered by drops also containing ethanol and glycerol, as excipients. Tifactor was recently approved in pediatric age as an oral solution, and does not contains ethanol.

The data from this study confirm the efficacy of both formulation in lowering TSH level and normalizing fT4 level within 15 days after starting therapy.

Currently, the initial recommended dose in CH for both Tirosint and Tifactor is the same (10-15 mcg/kg/day). We observed significant difference regarding the lowering of the TSH level after 15 days in subjects treated with the Tifactor solution compared to the newborns treated with the Tirosint drops, probably due to the slight higher bioavailability of Tifactor. No significant difference was observed in TSH level after 30 days and in fT4 levels at 15 and 30 days after initiation of treatment between the two groups, although subjects treated with Tifactor showed lower TSH level and higher fT4 level.

Tifactor dosage was lower with respect of Tirosint at diagnosis and after 15 and 30 days without significant statistical difference and with dosage titration based on TSH and fT4 levels in both cases.

In newborns treated with the Tifactor solution, we observed suppressed TSH level and higher than normal fT4 level in 55.6% of subjects at 15 and 30 days after the start of treatment. In newborns treated with Tirosint drops we observed higher than normal level of fT4 in 50% of subjects at 15 days and 25% of subjects at 30 days after the start of treatment, although these subjects showed a lower rate of TSH suppression (0/12 at 15 days and 1/12 at 30 days from the beginning of treatment).

Our data confirm the higher risk of overtreatment using liquid formulations, especially when using the Tifactor solution. This finding suggests possibly the need for a lower starting dose of L-thyroxine, when this formulation is used in newborns with CH (i.e. 7-12 mcg/kg/day).

The benefits of using Tifactor should consider the higher dosage targeting of L-thyroxine, as the minimal drug dosage change is 2 mcg, instead of 3.57 mcg when using Tirosint drops. Thus, it could be useful especially in preterm or low birth weight babies. Another factor to be considered is that Tifactor is an ethanol-free formulation, although Vigone et al. showed no side effects about growth or neurodevelopment using Tirosint drops in three-years follow-up study.

The limitations of this study are represented by the small size of the cohort and the short follow-up period. Larger cohorts and longer follow-up period are needed in further studies to determine better individualization of L-thyroxine dosage in newborns with CH and avoid the side effects of over- and undertreatment.

In conclusion, this study confirms the efficacy in normalizing the thyroid hormonal profile in newborns with CH among the liquid solutions although the response seem to be different in timing, therefore an individual approach is necessary considering the type of formulation used, the diagnostic category of CH and clinical features. Further studies in larger cohorts are needed to better determine the dose required for each of these formulations and to understand the possible influence of other factors on the different response to the therapy with the different liquid formulations.

## Data Availability Statement

The original contributions presented in the study are included in the article/Supplementary Material. Further inquiries can be directed to the corresponding author.

## Ethics Statement

The studies involving human participants were reviewed and approved by Ethics Committee of Health and Science City University Hospital of Turin. Written informed consent to participate in this study was provided by the participants’ legal guardian/next of kin.

## Author Contributions

GT and JM contributed to the study design, first draft of manuscript and statistical analysis. LS contributed to the study design, reference check and the revision of the final version of the manuscript. All authors contributed to the article and approved the submitted version.

## Conflict of Interest

The authors declare that the research was conducted in the absence of any commercial or financial relationships that could be construed as a potential conflict of interest.

The handling editor declared a past co-authorship with the authors.

## Publisher’s Note

All claims expressed in this article are solely those of the authors and do not necessarily represent those of their affiliated organizations, or those of the publisher, the editors and the reviewers. Any product that may be evaluated in this article, or claim that may be made by its manufacturer, is not guaranteed or endorsed by the publisher.
